# Development and Testing of an Aquaculture Environmental Control System Based on Behavioral Stress Responses

**DOI:** 10.3390/life15121809

**Published:** 2025-11-25

**Authors:** Bin Wang, Hang Yang, Hanping Mao, Qiang Shi

**Affiliations:** 1 School of Science and Technology, Shanghai Open University, Shanghai 200433, China; wangbin@sou.edu.cn; 2 School of Agricultural Engineering, Jiangsu University, Zhenjiang 212013, China; 3 Eco-Environmental Protection Research Institute, Shanghai Academy of Agricultural Sciences, Shanghai 201403, China; yanghangqu@foxmail.com

**Keywords:** stress behavior, control, aquaculture, crucian carp

## Abstract

This study addresses key challenges in intensive aquaculture, such as passive environmental control, high energy consumption, and neglected fish stress, through the development of a multi-objective environmental regulation system for crucian carp utilizing behavioral stress feedback. It combines YOLOv8s-FasterNet for behavior recognition, a specific growth rate model and an energy cost model to form an intelligent decision-making mechanism that maximizes the output–input ratio. In a 25-day experiment, the system showed strong performance. Final body weight and specific growth rate were comparable to the control group. Economically, the system achieved periodic profits that were 8.93, 1.43, and 1.03 times greater than those of traditional threshold control at external temperatures of 2 °C, 8 °C, and 14 °C, respectively, demonstrating significant energy savings. In terms of animal welfare, principal component analysis confirmed significantly lower stress-induced damage in the experimental group, with a comprehensive score (−0.036) closer to the initial healthy group (0.223) versus the control group (−0.348). These results indicate that the system successfully optimized both economic efficiency and fish health, providing a viable solution for intelligent aquaculture management.

## 1. Introduction

The continuous growth in global demand for high-quality animal protein has positioned aquaculture as a crucial player in ensuring food security [1,2]. In this context, intensive aquaculture has become the core direction of industrial development due to its high efficiency and high yield per unit area, and its production contribution accounts for a significant share of the total global aquaculture production [3,4]. Although high-density farming enhances production efficiency [5,6], it also significantly increases the risk of environmental deterioration within farming systems [7,8]. Key water parameters, particularly water temperature [9] and dissolved oxygen [10], are prone to severe fluctuations, thereby imposing chronic stress on cultured organisms [11]. Such environmental stress can trigger stress responses in fish [12], which not only suppress growth and development [13] and reduce feed utilization efficiency [14], but also compromise the immune system [15], leading to increased disease susceptibility [16]. Ultimately, these effects directly impact farming success and economic returns [17].

Among the various environmental parameters, water temperature and dissolved oxygen are two decisive controlling factors [18]. They not only individually affect the physiological metabolism [19] and behavioral activities [20] of fish, but also exhibit complex interactions [18]. For example, the rise in water temperature significantly increases the fish’s metabolic rate and oxygen consumption [21]. If the dissolved oxygen supply is insufficient at this time, it can easily induce severe combined stress, imposing a dual burden on the fish. Currently, the environmental control strategies in industrial aquaculture are mostly based on the fixed-threshold method [22], where the corresponding equipment is activated when sensors detect that a parameter falls below or exceeds a preset threshold. Although this method is simple and practical, it adopts a passive response mode, unable to proactively adapt to dynamic environmental changes. In addition, the unified threshold setting fails to fully consider the differentiated needs of fish at different growth stages and different circadian rhythms, as well as individual tolerance differences. Taking tilapia (*Oreochromis niloticus*) as an example, its optimal growth temperature during the seedling stage is about 30 °C, and as it grows, the optimal temperature gradually decreases [23]. The tolerance of juvenile rainbow trout (Oncorhynchus mykiss) to low dissolved oxygen is much lower than that of adult fish [24]. At the same time, the metabolic rate of fish fluctuates day and night, and their dissolved oxygen demand at night is usually lower than during the day [25]. A fixed dissolved oxygen threshold cannot adapt to this physiological rhythm. Currently, certain studies have endeavored to integrate intelligent control theory into the regulation of aquaculture environments. For instance, control systems grounded in fuzzy logic can establish rules based on expert knowledge to achieve a certain level of environmental adaptive adjustment. However, their rule bases are manually configured, making it challenging to handle complex and variable stress behavior feedback [26]. Furthermore, the Internet of Things (IoT) feedback system enables remote monitoring and threshold control, its regulatory approach remains passive, lacking real-time analysis and proactive intervention in fish behavioral states [27]. In contrast, the system proposed in this study utilizes fish stress behavior as a direct feedback signal, incorporating the YOLOv8s-FasterNet real-time recognition model and a growth-energy consumption multi-objective optimization mechanism. This facilitates a shift from “passive threshold control” to “active demand regulation,” offering enhanced dynamic adaptability and cost-effectiveness.

In recent years, with the advancement of the smart agriculture concept, behavior analysis technologies based on computer vision [28] have provided new possibilities for overcoming the aforementioned bottlenecks. When subjected to environmental stress, fish exhibit specific abnormal behavioral patterns [29]. These behaviors can serve as intuitive “early warning signals” of their physiological distress [30]. By identifying and analyzing these stress behaviors in real-time and using them as direct feedback for environmental regulation [31], it is possible to achieve an intelligent shift from “passive threshold-based control” to “active demand-based regulation,” thereby unifying aquaculture welfare and economic benefits. Regarding the selection of behavior recognition models, this study employs YOLOv8s-FasterNet as the core detection network, primarily due to its exceptional balance between accuracy and speed. Compared to other lightweight convolutional networks, such as the MobileNet series, which, despite their high computational efficiency, are prone to missed detections when identifying dense small targets (e.g., fish swarm behavior) [32]. EfficientDet, although excelling in general object detection, suffers from high model complexity, rendering it unsuitable for embedded deployment [33]. FasterNet reduces redundant computations through partial convolution (PConv), significantly decreasing both parameter count and inference latency while preserving high feature extraction capabilities, thereby making it more appropriate for real-time video stream processing.

To this end, this study aims to develop and validate a multi-objective control system for the aquaculture environment based on stress behavior feedback. Using crucian carp (Carassius auratus) as the research subject, the core work comprises the following: firstly, designing and constructing a comprehensive recirculating aquaculture hardware system. Secondly, establishing an improved YOLOv8s-FasterNet-based model for stress behavior recognition, a growth model quantifying the synergistic effects of temperature and dissolved oxygen on the specific growth rate, and a cost model for the energy consumption of control equipment operation. Finally, these models are integrated to formulate a multi-objective control strategy. This strategy aims to maximize the “output-input ratio” as its objective function, enabling the simultaneous optimization of fish growth performance, stress welfare, and energy consumption. Through systematic comparative farming experiments and an economic benefit analysis, this study verifies the effectiveness and superiority of the proposed strategy and system. The overarching goal is to provide innovative solutions and theoretical support for advancing the intelligence and sustainability of aquaculture.

## 2. Materials and Methods

The Animal Ethics Committee of the Shanghai Academy of Agricultural Sciences approved all animal procedures (SAASXM0625022).

### 2.1. Experimental System Configuration

**Figure 1:**
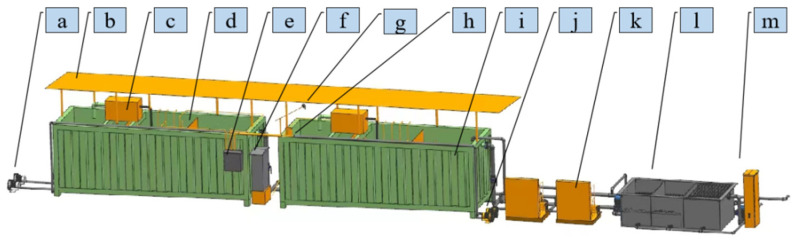
Recirculating Aquaculture System. (a) Aerator 1; (b) Sunshade net; (c) UV filter; (d) Partition net; (e) Low-voltage control cabinet; (f) High-voltage control cabinet; (g) Camera; (h) Waterproof acrylic barrel and laser emitter; (i) Container; (j) Aerator 2; (k) Water chiller; (l) biofilter; (m): Main control cabinet. The UV filter refers to a compact integrated unit for water filtration and oxygenation, equipped with an ultraviolet lamp that provides a certain degree of sterilization. The biofilter consists of a filter tank filled with filter media, which functions to adsorb impurities and cultivate nitrifying bacteria for water purification.

The multi-objective control system tested in this section was installed in a recirculating aquaculture system (RAS). The main components of the entire RAS are shown in Figure 1.

**Figure 2:**
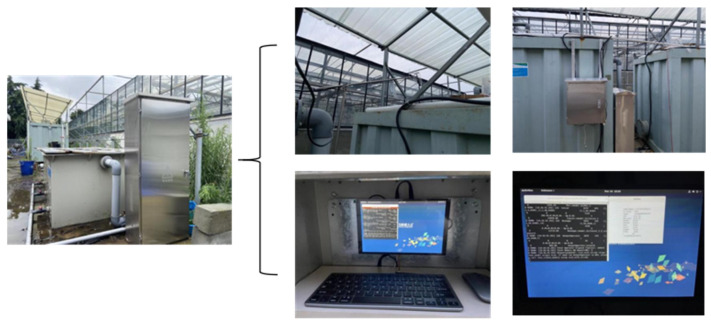
Photograph of the recirculating aquaculture system.

The system mainly consists of breeding containers, biological filters, and aeration devices. There are multiple aeration plates inside the breeding container. During operation, the water flows out from the bottom of the container under the action of gravity and first enters the biological filter with volcanic rock as the filter material. The porous structure of volcanic rocks provides a substrate for the growth of nitrifying bacteria. Through the nitrification of these bacteria, toxic substances such as ammonia nitrogen in water are converted into less toxic nitrates, thereby stabilizing water quality [34]. Subsequently, the water is pumped back to the container through a circulation pump. At the same time, a portion of the water flow will pass through a ultraviolet (UV) filter, which uses UV radiation to disinfect the water in real time to kill planktonic pathogenic microorganisms. The dissolved oxygen in the system is mainly provided and maintained by the aeration discs inside the container and the water drop oxygenation that may be formed during the reflux process. Finally, the treated water is returned to the aquaculture unit. The schematic diagram of the system structure is shown in Figure 2.

Once operational, the system could display information including water temperature, dissolved oxygen content, air temperature, types of abnormal fish behavior, and the optimal control strategy. Based on prior ethological research, this study defined surfacing and surface swimming of crucian carp as Hypoxic Stress Behavior 2. Additionally, loss of equilibrium was defined as Thermal Stress Behavior 1 [35]. A laser emitter in the system projected a red sheet laser to accurately identify the presence of crucian carp engaging in surface swimming.

### 2.2. Model Development

#### 2.2.1. Stress Behavior Recognition Model for Crucian Carp

Fifty healthy crucian carp were randomly selected, acclimated in the RAS, and introduced into the aquaculture tank. A laser emitter was fixed 5 cm above the water surface for planar scanning. Environmental interventions were applied through manual temperature variation and oxygen deprivation, and images were captured using a smartphone (resolution: 2160 × 1920 pixels). A total of 4680 original images were acquired, containing features corresponding to Behavior 1 and Behavior 2.

All images in the training set were annotated using Labellmg software 1.8.1 by drawing bounding boxes around the fish targets. To minimize background inclusion, annotations were based on the minimum bounding rectangle of each fish. The dataset was randomly split into training, validation, and test sets in an 8:1:1 ratio for model comparison. Training was conducted with a weight decay of 0.0005, an initial learning rate of 0.01, an input image size of 640 × 640, a batch size of 16, and 100 training epochs.

From the 4680 original images, 150 high-quality images were selected for model training. To enhance dataset diversity and model generalization while preventing overfitting, 13 data augmentation methods were applied using PyCharm 2024 and associated image processing libraries. These included random channel shuffling, brightness/contrast adjustment, flipping, grayscale conversion, scaling, translation, spot illumination, polygonal shading, rotation, perspective transformation, Gaussian blur, and cropping. Each of the 150 images was augmented using all 13 techniques, resulting in an augmented training set of 1950 images.

**Figure 3:**
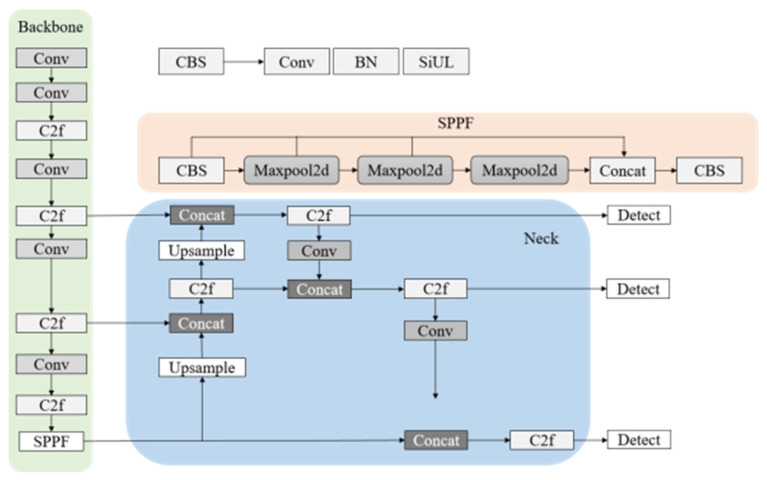
Architecture of YOLOv8s.

**Figure 4:**
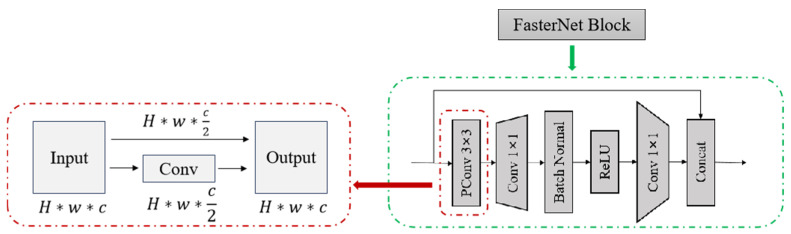
Basic Building Block of YOLOv8-FasterNet.

**Figure 5:**
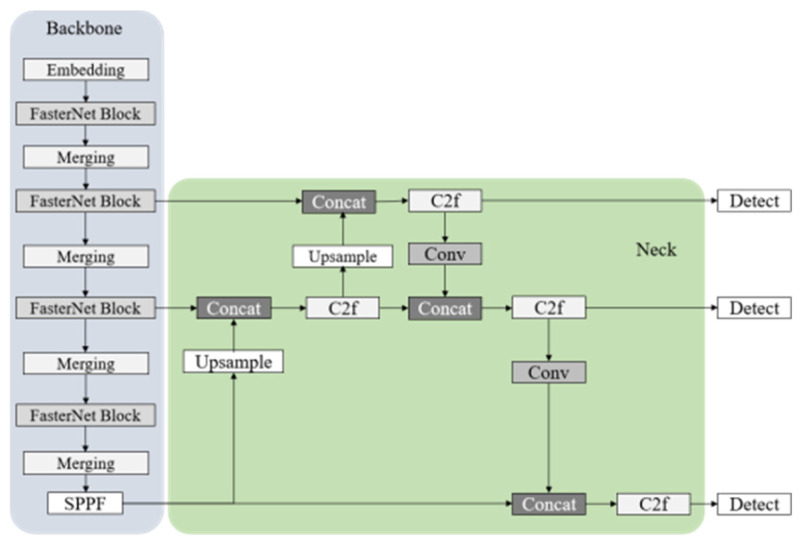
Overall Architecture of YOLOv8s-FasterNet.

The model was built upon the YOLOv8s architecture (Figure 3) and incorporated the lightweight neural network FasterNet as the feature extraction backbone (Figure 4). This design reduced the model size and enhanced inference speed. Features were extracted from the input image, and, after downsampling, prediction feature layers of varying sizes were generated. Subsequently, the model’s width was adjusted to half of the original T0 scale while maintaining its original depth, to further reduce the model size and improve inference speed. The training loss of the improved model followed the standard YOLO loss function. The overall structure of the YOLOv8s-FasterNet network is illustrated in Figure 5.

Furthermore, the evaluation metrics in this study included mean Average Precision (mAP), Precision (P), Recall (R), and the comprehensive evaluation metric F1-Score (F1). The calculation formulas are shown as (1)–(4). Generally speaking, a higher mAP indicates better detection performance. While mAP is an intuitive evaluation metric, a high mAP value alone cannot fully represent the overall performance. Therefore, the model’s parameter count and average detection speed were also introduced for a comprehensive assessment. The formulas for calculating mAP, Precision, Recall, and F1-Score were provided in the subsequent section.

(1) $$P=\frac{\mathrm{TP}}{\mathrm{TP}+\mathrm{FP}}\times 100\%$$

(2) $$R=\frac{\mathrm{TP}}{\mathrm{TP}+\mathrm{FN}}\times 100\%$$

(3) $$\mathrm{mAP}=\frac{1}{n}\sum_{i=1}^{n}{\mathrm{AP}}_{i}$$

(4) $$F_{1}\text{-}\mathrm{Score}=2\;\times \;\frac{P\times R}{P+R}\times 100\%$$

1. TP denoted positive samples that were correctly predicted as positive by the model.
2. FP denoted negative samples that were incorrectly predicted as positive by the model.
3. FN denoted positive samples that were incorrectly predicted as negative by the model.

#### 2.2.2. Specific Growth Rate Model for Crucian Carp

This study investigated the effects of temperature and dissolved oxygen (DO) on the specific growth rate (SGR) of juvenile crucian carp. A full factorial experimental design was employed, incorporating two factors: temperature (10, 20, 30 °C) and dissolved oxygen (2.5, 5.0, 7.5 mg/L). Each combination of factors was maintained for a 30-day period. A DO level of 2.5 mg/L was selected as the hypoxic condition based on the known tolerance of crucian carp to low oxygen, aiming to broaden the applicability of the model. To minimize confounding effects, the following conditions were maintained throughout the trial: a daily feeding ration equivalent to 2% of total fish biomass, a 30% daily water exchange, a pH of 7.0 ± 0.5, an ammonia nitrogen level of 0–0.12 mg/L, and a nitrite level of 0–0.12 mg/L.

A total of 300 experimental crucian carp (initial body weight: 48.9 ± 0.7 g) were sourced from the Linghu Aquaculture Base in Zhejiang Province. After a two-week acclimation period in a 1000 L tank, 270 fish were selected and randomly distributed into 27 aquaria (70 cm × 50 cm × 50 cm each), with 10 fish per aquarium. This setup provided three replicates for each of the nine experimental treatment groups. Based on the collected experimental data, a quadratic polynomial regression model describing the relationship of temperature and dissolved oxygen with the specific growth rate was developed using MATLAB software 2016b.

#### 2.2.3. Environmental Control Cost Model

The multi-objective control strategy of this study aimed to achieve intelligent regulation by coordinating multiple dimensions, including environmental conditions, fish growth performance, equipment power consumption, and cost. This strategy operated by predicting water parameters and operational costs under different control schemes. These predictions were integrated with growth performance data and stress feedback following temperature and oxygen stress. The optimization goal was to maximize the output-input ratio, thereby creating an economical and suitable growth environment for crucian carp and ultimately enhancing the economic efficiency of industrial aquaculture. To achieve precise control of water temperature within the container-based aquaculture environment, a power consumption calculation model was developed in this section.

- Heating Power Consumption Model

The water volume was set to be 8000 kg in the container, the current air temperature was set to be $T_{1}$, and the target water temperature was set to be $T_{2}$. The total power of the heating rods ($P_{z}$) was 38 kW. Taking heat dissipation loss into account, the calculation formulas of the actual heating power are shown as (5)–(7).

(5) $$P_{j}=P_{z}-P_{s}$$

$P_{j}$ denoted the actual heating power of the heating rods, in kilowatts (kW);
$P_{z}$ denoted the total installed power of the heating rods, in kilowatts (kW);
$P_{s}$ denoted the total heat dissipation power during the heating process, in kilowatts (kW).

The total heat loss $P_{s}$ comprised heat dissipation from the container itself ($P_{\mathrm{xs}}$) and heat loss from the water body ($P_{\mathrm{ss}}$), both calculated according to Newton’s Law of Cooling:

(6) $$P_{j}=P_{z}-17\;\times \left(T_{2}-T_{1}\right)\times 12-40\;\times \left(T_{2}-T_{1}\right)\times \;22.72$$

The heat dissipation areas were determined experimentally as $S_{\mathrm{js}}=$ 22.72 m^2^, $S_{\mathrm{ss}}\;$= 12 m^2^ ($S_{\mathrm{js}}$, the heat dissipation area of the water body to the container; $S_{\mathrm{ss}}$, the heat dissipation area of the water body to the air). Substituting these values yields:

(7) $$P_{j}=38,000-1112.8\times \left(T_{2}-T_{1}\right)$$

$T_{2}$ denoted the target water temperature during the heating process, °C;
$T_{1}$ denoted the temperature of the container during the heating process, °C.

- Cooling Power Consumption Model

The total refrigeration capacity of the chiller was $P_{\mathrm{zz}}$ = 27 kW. Considering a heat loss coefficient of 0.9, which was an empirical coefficient, the calculation formulas of the effective refrigeration capacity are shown as (8)–(9).

(8) $$P_{\mathrm{zl}}={0.9P}_{\mathrm{zz}}-P_{\mathrm{zs}}$$

The heat dissipation loss $P_{\mathrm{zs}}$ was calculated using the same method as applied in the heating process, and was ultimately simplified to the following expression:

(9) $$P_{\mathrm{zl}}=24,300-1112.8\times \left(T_{1}-T_{2}\right)$$

- Aeration Control Model

This experiment was designed to investigate the effect of water temperature on the oxygenation rate of the aerator. The trial was conducted within an aquaculture container, where the water depth was maintained at 0.4 m to reduce the total water volume, promote oxygen consumption, and thereby enhance experimental efficiency. Although the water depth was shallower than typical production systems, the core of this model was to establish a functional relationship between temperature and oxygenation rate. The oxygenation rate mainly depended on the performance of the oxygenation equipment, and the physical characteristics of the water body has no direct functional relationship with water depth. The recirculating water control system was used to regulate the water temperature, while sensors were employed to monitor the water temperature and dissolved oxygen levels in real-time.

Six temperature gradients were established: 13, 15, 17, 19, 21, and 23 °C. Experimental timings were flexibly scheduled based on weather forecasts, and heating rods were used cooperatively to adjust water temperature for energy conservation. Prior to testing, a total of 300 juvenile crucian carp were used in the system validation experiment. The fish were sourced from the Linghu Aquaculture Base in Huzhou City, Zhejiang Province, China. The average body weight was 51.8 ± 3.5 g, with a total length ranging from 12 to 15 cm. The test commenced once the dissolved oxygen level decreased to approximately 6 mg/L. The aerator was then activated, and the time required for the dissolved oxygen to increase from 6 mg/L to 8 mg/L was recorded. The average oxygenation rate was calculated based on this duration. Each temperature condition was tested in triplicate. The relationship between water temperature x and oxygenation rate y was established through experimental fitting. The calculation formula is shown as (10).

(10) y=0.36×10−x8.17 +0.18

y denoted the oxygenation rate of the aerator, mg/L·min;
x denoted the temperature of the water body in the container, °C.

- Development of the Control Cost Model

Control costs were categorized into intermittent control and continuous control, and calculated based on the market electricity price $C_{1}$ (RMB/kW·h).

1. Intermittent Control Cost:

The primary equipment for intermittent control was the laser emitter. Its working principle was to project a red sheet-like laser onto the water surface, forming a detection light curtain. When crucian carp swam on the surface, they would pass through this light curtain and be accurately recognized by the system. To avoid continuous interference with fish and reduce energy consumption, the laser was not continuously turned on, but operated in intermittent mode. After pre-experimental optimization, the working duty cycle was set to 40%, which meant running for 0.4 h per hour at a power of $N_{0}$ = ${5\times 10}^{-4}$ kW. This setting could maximize energy efficiency while ensuring behavior capture rate. The calculation formula is shown as (11).

(11) $$E_{0}=0.4\times N_{0}\times C_{1}$$

$E_{0}$ denoted the hourly cost of operating the laser emitter, kW·h;
$N_{0}$ denoted the power rating of the laser emitter, in kilowatts kW.

2. Continuous Control Cost:

This category included equipment such as heating rods, chillers, water pumps, industrial cameras, and sensors. Their operating durations were associated with regulation processes such as temperature control and oxygenation. The calculation formula is shown as (12).

(12) $$E_{n}=P_{n}\times N_{n}\times C_{1}$$

$E_{1}–E_{7}$ denoted the hourly energy consumption of the respective continuous control devices, kW·h;
$N_{1}–N_{7}$ denoted the power ratings of the respective continuous control devices, kW.

3. Objective Function

This study employed the Output-Input Ratio maximization as the objective function. The optimal control strategy, in terms of economic benefit, was selected by comparing the $D_{n}$ values across different temperature and oxygen control schemes. The nighttime strategy appropriately “cooled and increased oxygen” relative to the daytime baseline to enhance the stress resistance of the fish. The calculation formula is shown as (13).

(13) $$D_{n}=\frac{F_{n}}{E_{n}}$$

$F_{n}$ denoted the value increment of the crucian carp over the cycle, RMB;
$E_{n}$ denoted the total cost of aquaculture environmental control during the cycle, RMB.

### 2.3. Multi-Objective Control Strategy

**Figure 6:**
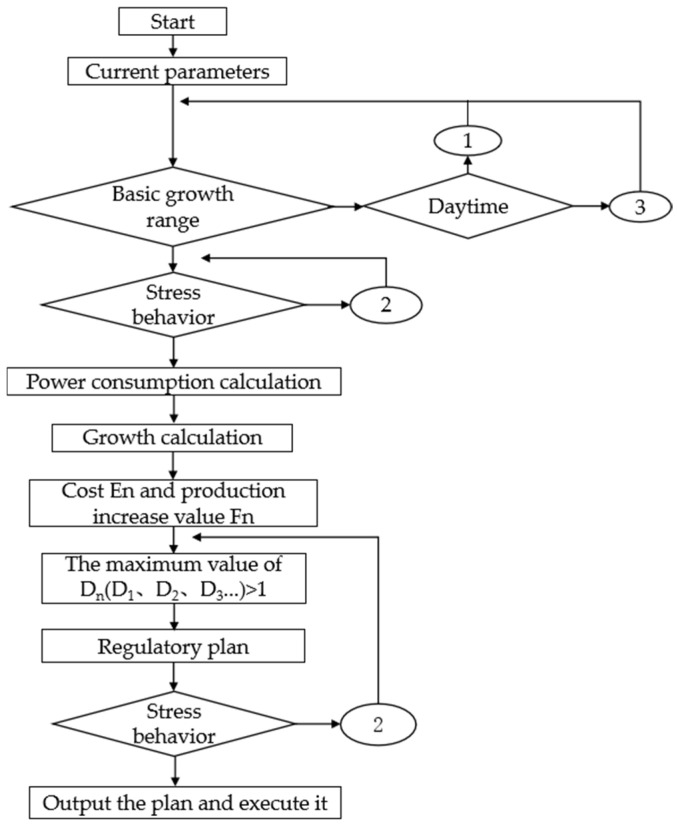
Control flow for economically optimal objectives. The figure shows the overall control logic of the system in daytime mode, with the goal of maximizing the input-output ratio. The system first obtained current water temperature and dissolved oxygen data, combined with feedback on fish stress behavior, and predicted the economic benefits under different control schemes through multiple model calculations (growth model, energy consumption model). Finally, the temperature oxygen combination with the highest benefit was selected as the execution strategy to achieve coordinated optimization of economy and welfare. Among them, process 1 was based on daytime regulation, process 2 was based on stress behavior regulation, and process 3 was based on nighttime regulation, which was similar to process 1 but differed in temperature and oxygen range.

To ensure that crucian carp consistently grow under suitable and cost-effective environmental conditions, a multi-model calculation approach was employed to identify the optimal combination of temperature and oxygen parameters that yielded the highest output-to-input ratio. Specifically, by comparing the $D_{n}$ values across different schemes, the one with the maximum $D_{n}$ was selected as the execution plan. The detailed control process is illustrated in Figure 6.

During daytime, the aquaculture environment control strategy used ambient temperature as the baseline and incorporates the temperature–oxygen synergy to promote fish growth. Oxygen levels were dynamically adjusted based on behavioral feedback from the fish (e.g., side rolling as stress behavior 1; surfacing and surface swimming as stress behavior 2), aiming for economically optimal control while avoiding significant stress responses. Based on the findings from our preliminary studies, the temperature was regulated within the range of 10–30 °C [35], and dissolved oxygen was maintained no lower than 5 mg/L, without exceeding the saturated dissolved oxygen level at the current water temperature (e.g., 10.08 mg/L at 15 °C). The system selected and implemented the parameter combination within the feasible temperature–oxygen domain that yielded the highest economic benefit.

**Figure 7:**
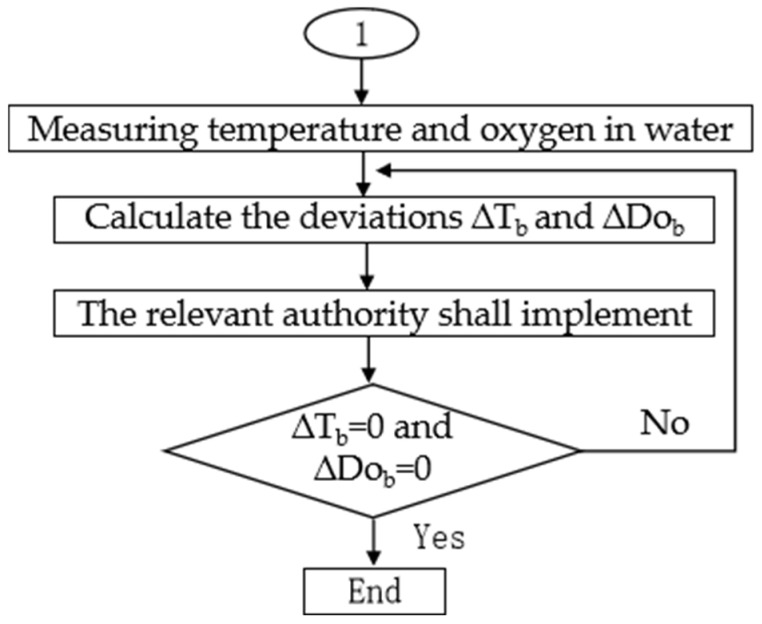
Control strategy process one. This process was applicable when the initial environmental conditions could not meet the basic survival needs of the fish population. The system prioritized adjusting the water temperature to the appropriate range (10–30 °C), and then adjusting the dissolved oxygen to the minimum survival standard (≥5 mg/L). In the figure, ΔT_b_ and ΔDo_b_ respectively represent the deviation between the current value and the target threshold, and the system determined the priority and amplitude of heating/cooling or oxygenation based on this.

Control Process 1 was applied when the initial temperature and dissolved oxygen conditions in the aquaculture environment could not meet the basic survival requirements of crucian carp. The specific steps are shown in Figure 7. Here, $∆T_{b}$ denoted the difference between the current water temperature and the upper or lower limit of the suitable temperature range for crucian carp, while $∆{\mathrm{Do}}_{b}$ represented the difference between the current dissolved oxygen level and the basic oxygen demand of crucian carp.

**Figure 8:**
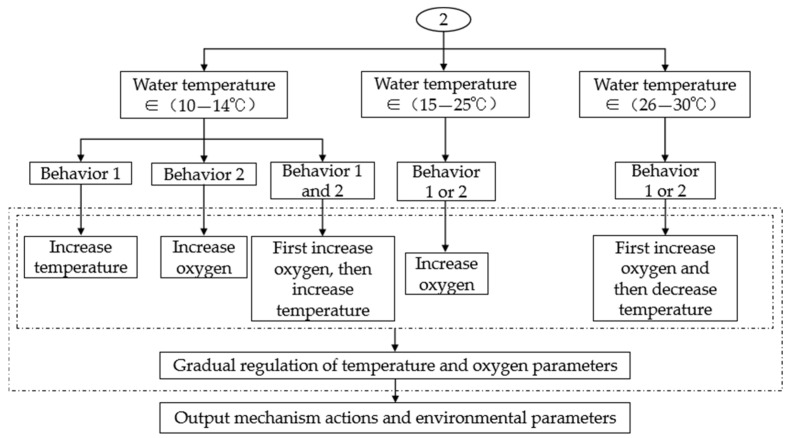
Control strategy process two. This process implemented differentiated regulation based on different water temperature ranges and types of fish stress behavior. When in the low temperature range (10–14 °C), the system prioritized oxygen supplementation to alleviate the hypoxia behavior of crucian carp, and if necessary, raised the temperature. When in the appropriate range (15–25 °C), the system prioritized oxygenation when crucian carp exhibit any stress behavior. When in the high temperature range (26–30 °C), the system prioritized oxygenation first, and if it failed, the cooling program was initiated. By dynamically regulating in stages and types, the system ensured the health of the fish population while minimizing energy consumption.

As shown in Figure 8, Control Process 2 adopted corresponding regulation strategies based on the type of crucian carp stress behavior and the current water temperature range:

- When water temperature was 10–14 °C

If stress behavior 1 occurred, the temperature was gradually increased (by +1 °C per step) until the upper limit was reached, after which oxygen augmentation was initiated. If stress behavior 2 occurred, oxygen was gradually increased (by +1 mg/L per step) up to the saturated dissolved oxygen level. If both stress behaviors occurred simultaneously, oxygen was prioritized until behavior 2 disappeared, after which heating was considered.

- When water temperature was 15–25 °C

Upon occurrence of any stress behavior, oxygen was increased preferentially and progressively (by +1 mg/L per step) until the behavior ceased.

- When water temperature was 26–30 °C

If stress behavior occurred, oxygen was increased stepwise as the first response. If the behavior persisted after reaching the oxygen upper limit, cooling was activated (by −1 °C per step) until the behavior disappeared.

Moreover, the aquaculture environment cannot rely on fish stress behavior for feedback regulation during the nighttime. Studies have shown that controlling the rate of water temperature change within a reasonable range at night can enhance disease resistance in fish, thereby helping to prevent and alleviate stress [36]. Within the normal temperature range, an hourly temperature variation not exceeding 4–5 °C has been shown to cause no adverse effects on fish [37]. Based on this, under normal regulation conditions, this study set the lower temperature limit at night to the evening entry temperature minus 4 °C, while other control measures remained the same as those applied during the daytime.

### 2.4. Design of the System Validation Experiment

To evaluate the effects of the multi-objective regulation system on the growth and physiological state of juvenile crucian carp, a farming trial was conducted based on the SGR model. The water depth of the experimental tanks was 0.67 m, and the water was pretreated with 48 h of aeration. Key water quality parameters were maintained as follows: ammonia nitrogen below 0.12 mg/L, nitrite below 0.02 mg/L, and pH at 7.00 ± 0.50. A total of 300 juvenile crucian carp (average weight approx. 51.8 g) were introduced from the Linghu Base in Huzhou. They were divided into a regulated group and a threshold group for a two-week acclimation period. During acclimation, one-third of the water was changed daily, commercial feed was provided at 2% of the total fish body weight, and facial laser pre-adaptation was applied to reduce stress.

After acclimation, 10 fish were randomly selected as the initial group. Serum was collected from these fish to determine physiological indices—including lysozyme (LZM), superoxide dismutase (SOD), catalase (CAT), malondialdehyde (MDA), and cortisol—to establish baseline data. The remaining fish were then randomly allocated to the experimental group and the threshold group (20 °C, 5 mg/L) for a 25-day feeding trial.

At the end of the farming period, 10 fish from each group were fasted for 24 h, anesthetized using MS-222, and weighed. Blood samples were collected via caudal puncture and allowed to clot for 1 h. The samples were then centrifuged at 2000× *g* for 10 min to separate the serum. The aforementioned physiological indicators were measured using commercial assay kits.

### 2.5. Data Analysis

Data analysis was performed using GraphPad Prism 5.0 software. All data were first subjected to normality test using Shapiro–Wilk test, and then subjected to homogeneity of variance test using F-test. After confirming that the data met the assumptions of parameter testing, intergroup comparisons were conducted using unpaired t-tests, while intragroup differences were assessed by one-way analysis of variance (ANOVA). Statistical significance was defined as *p* < 0.05. All results were expressed as mean ± standard deviation. In addition, PCA was used for dimensionality reduction analysis of five standardized stress physiological indicators (LZM, SOD, CAT, Cortisol, and MDA). This method was implemented using GraphPad Prism 5.0 software to extract principal components that reflected most of the original data variations, and calculate the comprehensive scores of each experimental group for ranking comparison.

## 3. Results

### 3.1. Analysis of Stress Behavior Detection Results by YOLOv8s-FasterNet

The analysis of detection results for different stress behaviors in crucian carp, using the YOLOv8s-FasterNet model, is presented in Table 1.

As shown in Table 1, the YOLOv8s-FasterNet model demonstrated excellent recognition performance for the three stress behaviors in crucian carp, achieving mAP, P, R, and F1 of 98.23%, 98.20%, 95.30%, and 96.72%, respectively. The high F1-Score of 96.72%, a comprehensive metric, indicated the model’s outstanding overall capability for reliable stress behavior detection.

### 3.2. Effect of Temperature and Oxygen on the Specific Growth Rate of Juvenile Crucian Carp

The parameters in the regression equation were determined using the least squares method. Based on the data from the full factorial experiment, regression analysis was performed to obtain the regression equation and response surface [38].

**Figure 9:**
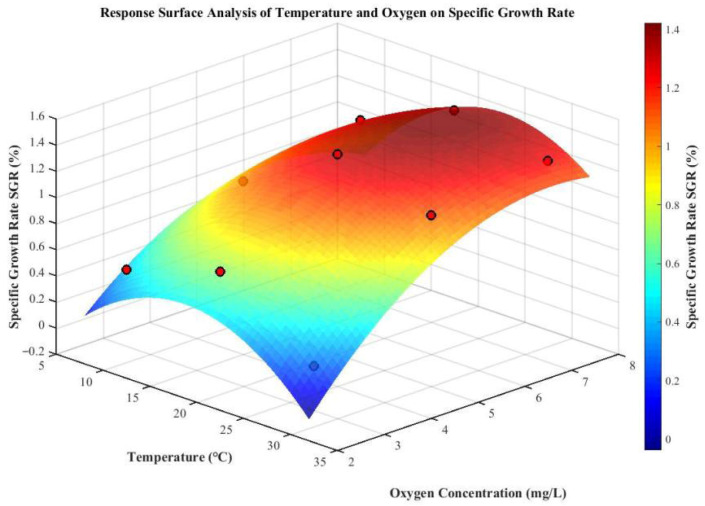
Response surface plot of the effects of temperature and oxygen on the SGR of juvenile crucian carp.

The influence of temperature and oxygen levels on the specific growth rate (SGR) of juvenile crucian carp is shown in Figure 9. The least squares method was applied to perform regression fitting on the data. The calculation formula is shown as (14).

(14) $$\mathrm{SGR}=-1.355+0.108A+0.479B+4.3\times 10^{-3}\mathrm{AB}-3.125\times 10^{-3}A^{2}-0.043B^{2}$$

A represented temperature, °C;
B denoted oxygen concentration, mg/L.

Analysis of variance indicated that the regression model was highly significant (*p* < 0.01), with a coefficient of determination (R^2^) of 0.97, demonstrating a good fit and supporting its use for predicting the SGR of juvenile crucian carp. Both temperature and dissolved oxygen had significant effects on SGR (*p* < 0.05), and a significant interaction was observed between the two factors (*p* < 0.05).

As shown in the response surface plot in Figure 9, within the experimental range, SGR initially increased and then decreased with rising temperature and dissolved oxygen levels. The maximum SGR of 1.36% was achieved at 20 °C and 7.5 mg/L. Under sufficient dissolved oxygen conditions (5–7.5 mg/L), low temperature (10 °C) exerted a stronger inhibitory effect on SGR than high temperature (30 °C), primarily due to the significant suppression of feeding behavior at low temperatures. In contrast, under hypoxic conditions (2.5 mg/L), high temperature (30 °C) showed a stronger inhibitory effect on SGR, likely because elevated temperatures exacerbate dissolved oxygen deficiency, further restricting fish growth. Moreover, at the same temperature, high oxygen levels (7.5 mg/L) promoted SGR more effectively than normal oxygen levels (5.0 mg/L), which in turn outperformed low oxygen conditions (2.5 mg/L).

### 3.3. Changes in Serum LZM Levels of Crucian Carp Under Different Treatments

**Figure 10:**
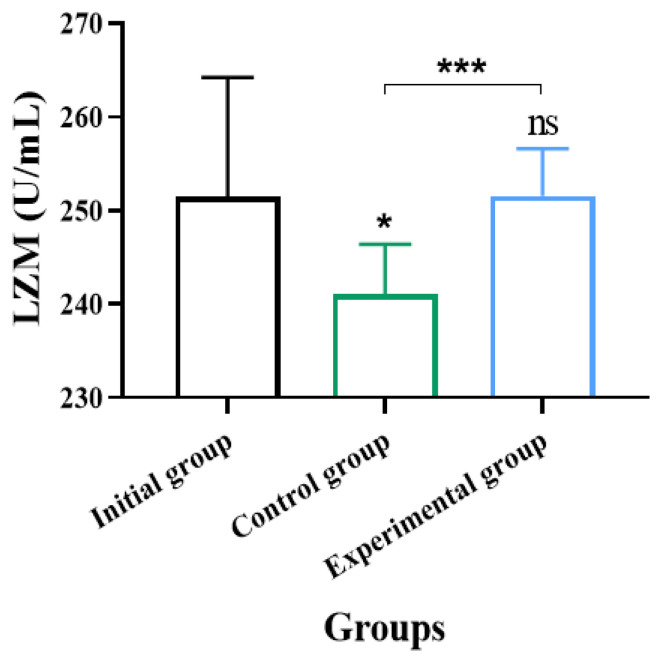
Effect of multi-objective regulation strategy on crucian carp LZM. * indicated *p* < 0.05; *** indicated *p* < 0.001; ns indicated no significant difference, n = 9.

Changes in serum LZM activity of crucian carp under different aquaculture environments are shown in Figure 10. The LZM activity in the control group was significantly lower than that in the initial group and the experimental group, with reductions of 4.16% and 4.14%, respectively (*p* < 0.05, effect size = 0.47, 95% CI [−19.56, −1.24]). No significant difference was observed between the experimental and initial groups, although the experimental group showed a slightly higher value. This result may be attributed to the better aquaculture environment maintained by the multi-objective regulatory system. The system effectively reduced the chronic stress level of fish by dynamically optimizing the water environment and intervening in a timely manner based on stress behavior, which may be the reason why the LZM activity in the experimental group was maintained at a high level.

### 3.4. Effects and Analysis of the Multi-Objective Regulation Strategy on Growth Parameters of Crucian Carp

The effects of different aquaculture environment regulation schemes on the final weight (FW), weight gain rate (WGR), SGR and survival rate (SR) of crucian carp are shown in Table 2.

The control group exhibited higher values in FW, WGR, and SGR compared to the experimental group, with increases of 2.72%, 8.89%, and 8.08%, respectively. These differences were not statistically significant. In addition, since the final weight range of crucian carp in both the experimental and control groups still fell within the juvenile stage, variations in physiological parameters due to body weight were minimal.

### 3.5. Effects and Analysis of the Multi-Objective Regulation Strategy on Physiological Parameters of Crucian Carp

**Figure 11:**
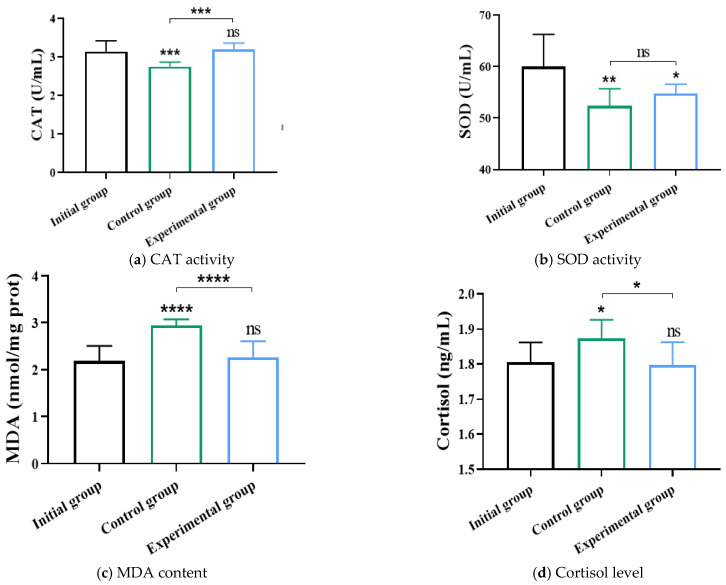
Effect of multi-objective regulation strategy on stress related parameters of crucian carp. * indicated *p* < 0.05; ** indicated *p* < 0.01; *** indicated *p* < 0.001; **** indicated *p* < 0.0001; ns indicated no significant difference, n = 9.

As shown in Figure 11a–d, in the comparison between the experimental and control groups, the control group exhibited significantly lower serum CAT activity (a reduction of 13.85%, *p* < 0.0001, effect size = −0.83, 95% CI [0.30, 0.58]), significantly higher MDA content (an increase of 34.97%, *p* < 0.0001, effect size = 0.797, 95% CI [−0.92, −0.43]), and significantly higher cortisol levels (an increase of 4.04%, *p* < 0.05, effect size = 0.54, 95% CI [−0.13, −0.02]) compared to the experimental group. In contrast, no significant difference was observed in SOD activity between the two groups. Both CAT and SOD are important antioxidant enzymes that work synergistically to scavenge free radicals and mitigate oxidative damage. Although fish in both groups experienced a certain degree of environmental stress after being transferred to new conditions and eventually stabilized, the differential responses in CAT and SOD activities may be related to individual nutritional status or sampling variability. Collectively, the higher levels of MDA and cortisol in the control group suggested a greater degree of oxidative stress compared to the experimental group.

In the comparison between the experimental and initial groups, the experimental group showed significantly lower SOD activity (a decrease of 4.61%, *p* < 0.05, effect size = −0.5, 95% CI [−9.6, −0.9]) than the initial group, while no significant differences were observed in CAT activity, MDA content, or cortisol levels. Given the functional similarity between SOD and CAT in antioxidant defense, it was appropriate to evaluate the stress status comprehensively by integrating indicators such as MDA and cortisol. MDA served as a marker of lipid peroxidation, and cortisol was a typical stress hormone; higher levels of both generally reflect stronger stress responses. The absence of significant differences in these two indicators between the experimental and initial groups indicated that the stress level experienced by the experimental group was comparable to that of the initial group.

### 3.6. Economic Benefit Analysis

This study aimed to develop a multi-objective regulation strategy and system to balance fish growth performance with aquaculture energy consumption, improving economic efficiency while effectively controlling stress levels in fish. Therefore, this section theoretically compared the equipment energy consumption and farming profitability between the multi-objective strategy and conventional strategies under different environmental conditions.

The experimental group adopted a multi-objective regulation strategy based on stress behavior feedback, while the control group used a threshold control strategy commonly applied in industrial aquaculture-maintaining a constant temperature of 20 °C and a dissolved oxygen level of 5 mg/L. Research indicated that temperature control was the primary source of energy consumption in aquaculture, particularly in outdoor intensive container-based farming models. Apart from the difference in temperature control strategies, all other equipment remained consistent between the experimental and control groups, with negligible differences in power usage. Thus, the disparity in energy consumption primarily stemmed from temperature regulation.

Based on the temperature characteristics of early December in Jiangsu Province (2–14 °C, average 8 °C), this study selected three typical ambient temperatures (2 °C, 8 °C, and 14 °C) to simulate the energy consumption of the experimental group regulating within the 10–30 °C range and the control group maintaining a constant 20 °C. To reflect real high-density aquaculture conditions, the water mass was set at 8000 kg and the total fish biomass at 300 kg for quantitative analysis of energy consumption and profit.

Under an ambient temperature of 2 °C, the energy consumption and profit of the experimental and control groups are shown in Table 3.

As shown in Table 3, when the ambient temperature was 2 °C, the energy consumption cost of the experimental group increased with the rise of the regulated temperature. When the target temperature was 10 °C, the energy cost was 48.76 RMB; when the temperature increased to 20 °C, the cost reached 177.63 RMB, which was comparable to that of the control group. The profit analysis revealed better economic performance: the experimental group achieved the highest profit of 47.78 RMB at 15 °C, which was 8.93 times that of the control group (5.35 RMB). Therefore, under these conditions, implementing Scenario 6 (regulation to 15 °C) was the economically optimal choice.

According to Table 4, when the ambient temperature was 8 °C, the energy consumption cost of the experimental group increased with the rise in the regulated temperature. At a target temperature of 10 °C, the energy consumption was 9.91 RMB; when the temperature increased to 20 °C, the cost reached 86.34 RMB, which was the same as that of the control group. At this point, the profit of the control group was 96.64 RMB. The profit of the experimental group was lower than that of the control group until 13 °C, after which it first increased and then decreased, peaking at 17 °C with a value of 110.55 RMB. This peak profit was 1.43 times that of the control group. When the temperature exceeded 17 °C, the profit gradually declined, and losses occurred beyond 25 °C. Therefore, at an ambient temperature of 8 °C, implementing Scenario 8 (regulation to 17 °C) in the experimental group represented the economically optimal choice.

According to Table 5, when the ambient temperature was 14 °C, the energy consumption cost of the experimental group increased with the rise in the target temperature. At a target temperature of 14 °C, the energy consumption cost was 0 RMB; when the target temperature increased to 20 °C, the cost reached 33.97 RMB, which was the same as that of the control group. Under these conditions, the profit of the control group was 149.02 RMB. The profit of the experimental group was lower than that of the control group until 16 °C, after which it first increased and then decreased, peaking at 18 °C with a value of 153.88 RMB. This peak profit was 1.03 times that of the control group. When the temperature exceeded 18 °C, the profit gradually declined, and losses occurred beyond 28 °C. Therefore, at an ambient temperature of 14 °C, implementing Scenario 5 (regulation to 18 °C) in the experimental group represented the economically optimal choice. Integrating the results under the three ambient temperature conditions (2 °C, 8 °C, and 14 °C), the multi-objective regulation system consistently yielded higher profits than the control group during the heating process, with profits being 8.93 times, 1.43 times, and 1.03 times those of the control group, respectively. This demonstrated a clear economic advantage of the proposed system.

While the economic benefits reported in this study were based on specific climatic and economic conditions in southern China, the proposed multi-objective control system possessed inherent adaptability. The core framework—integrating behavior recognition, growth modeling, and cost calculation—was universal. For implementation in new geographical regions, the model can be readily re-parameterized with local data, including regional temperature profiles, local electricity costs, and market prices. This ensures that the system retains its capability to identify the most economically efficient control strategy tailored to any specific aquaculture environment.

The electricity price adopted in this study referenced the typical industrial electricity tariff in Shanghai, China, providing reasonable regional representativeness. The water volume was set to 8000 kg based on the actual capacity of the aquaculture container used in the experiment. Although actual electricity prices may exhibit time-of-use or seasonal fluctuations, a static electricity price assumption was applied in this study to simplify the modeling framework. Should a dynamic pricing mechanism be incorporated in the future, the economic performance of the system could be further optimized, particularly by scheduling energy-intensive control operations during low-tariff periods.

### 3.7. Comprehensive Assessment of Stress-Induced Damage

**Figure 12:**
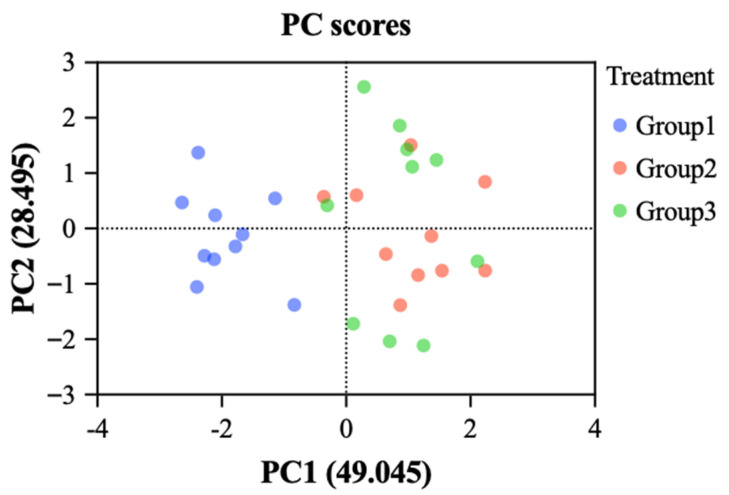
PCA plot was used for screening and categorization of influencing factors. Group 1, initial group; Group 2, control group; Group 3, experimental group.

In this experiment, five stress-induced damage indicators in fish—LZM, SOD, CAT, cortisol, and MDA levels—were selected for screening and categorization of influencing factors using principal component analysis (PCA). The KMO test and Bartlett’s test of sphericity indicated that the data were suitable for factor analysis. Two principal components (k = 2) with eigenvalues greater than 1 were extracted, having eigenvalues of 2.452 and 1.425, respectively. These components accounted for variance contribution rates of 49.045% and 28.495%, respectively, with a cumulative variance contribution rate of 77.540% (Figure 12 and Table 6).

Each principal component could be expressed as a linear combination of the five indicators, with only a few indicators exhibiting high loadings. Larger loadings indicated stronger correlations with the principal component. As shown in Table 6, the first principal component (F_1_), which contributed 49.045% of the variance, had loadings greater than 0.351 for SOD activity, CAT activity, and MDA content, indicating that F_1_ primarily reflected information from these three indicators. The second principal component (F_2_), with a contribution rate of 28.495%, had loadings greater than 0.488 for LZM activity and cortisol concentration, suggesting that F_2_ mainly represented the characteristics of these two indicators. The principal component loading matrix values in Table 7 were further divided by the square root of the corresponding initial eigenvalues to obtain the principal component coefficient matrix. Each column of this matrix represented an eigenvector, which was used to construct the linear combination expression of the preprocessed variables. The calculation formula is shown as (15) and (16).

(15) $$F_{1}=-0.057X_{1}+0.307X_{2}\;+0.241X_{3}+{0.032X}_{4}-0.224X_{5}$$

(16) $$F_{2}=-0.445X_{1}+0.239X_{2}-0.05X_{3}+{0.409X}_{4}+{0.107X}_{5}$$

Combining the variance values of F_1_ and F_2_ in Table 6, the comprehensive principal component model for fish stress damage, denoted as F, can be derived as a function of F_1_ and F_2_. The calculation formula is shown as (17)–(19).

(17) $$F=\frac{49.045}{77.540}F_{1}+\frac{28.495}{77.540}F_{2}$$

The model can be expressed as follows:

(18) $$F=0.632F_{1}+0.368F_{2}$$

Based on the above Equations, the following results can be derived:

(19) $$F={-0.2X}_{1}+{0.282X}_{2}+{0.134X}_{3}+{0.171X}_{4}-{0.103X}_{5}$$

Based on the comprehensive principal component model, the composite principal component values can be calculated. By sorting these values, the stress-induced damage status of the experimental fish can be evaluated. A composite score closer to that of the initial group indicated a lower degree of stress-induced damage. Therefore, the standardized mean values of the test result from the initial group, experimental group, and control group were substituted into Equation (19), respectively. The results are shown in Table 8. As presented in Table 8, Group A exhibited the lowest degree of stress-induced damage. The composite score of Group B was the closest to that of Group A, while Group C showed the poorest performance. This indicated that the stress-induced damage in crucian carp from the experimental group was less severe than that in the control.

## 4. Discussion

The effectiveness of the multi-objective regulation system developed in this study stems from the synergistic operation and accurate prediction of its three core models. First, the stress behavior recognition model based on the improved YOLOv8s-FasterNet enabled early, non-contact diagnosis of fish discomfort, providing critical real-time feedback signals for the system. Second, the specific growth rate model established through full-factorial experiments accurately quantifies the combined effects of temperature and dissolved oxygen on the growth of crucian carp, offering a theoretical basis for achieving optimal growth performance. Finally, the dynamic cost model, which integrated heating, cooling, and oxygenation processes, translates environmental regulation into clear economic indicators. By integrating these models and optimizing them under the unified objective function of maximizing the “output-input ratio,” the system overcame the limitations of traditional threshold-based control, effectively balancing growth performance, animal welfare, and economic efficiency.

The method of PCA has been widely applied in fisheries research, demonstrating effectiveness in comprehensive evaluations such as fish quality, freshness, and mechanical damage [39]. Unlike unsupervised learning methods such as cluster analysis, the core objective of PCA is not to group samples, but to reveal the intrinsic structure between the original variables by constructing a few unrelated principal components and calculating the comprehensive score of each sample. This comprehensive score can compress multidimensional data information into one dimension, allowing us to quantitatively rank and compare the degree of stress damage in different groups. In contrast, cluster analysis focuses more on discovering unknown natural categories in the data and is not suitable for directly verifying the degree of differences between known groups [40]. Previous studies often relied on single-factor indicator analysis, which, while straightforward to implement, struggled to fully capture the overall changes in the subject of study. To address this, the present study selected five stress-related parameters in fish—LZM, SOD, CAT, cortisol and MDA level—and employed PCA to comprehensively assess the impact of the multi-objective aquaculture environment regulation system on stress-induced damage in crucian carp. By extracting correlations among parameters and eliminating redundant information, the original five parameters were reduced to two principal components, with a cumulative contribution rate of 77.540%. This indicated that these two principal components encapsulate most of the information from the original indicators. The composite scores derived from them can be used to rank stress damage levels, confirming the feasibility and objectivity of PCA in this evaluation while improving the efficiency of data analysis.

Studies have shown that fish serum parameters can reflect their non-specific immune capacity, and related enzyme activities serve as important indicators for assessing stress status [41,42]. As a primary bactericidal substance, LZM plays a key role in resisting pathogen invasion [43], and variations in its activity can indicate the level of external stress. In this study, the serum LZM activity of crucian carp in the experimental group was significantly higher than that in the control group. This enhancement in innate immunity suggested that the multi-objective regulation system effectively mitigated chronic stress. Rather than statically maintaining a single ‘optimal’ temperature, the system dynamically managed the thermal environment based on real-time fish behavior and economic objectives. This approach likely prevented the fish from being exposed to prolonged periods of sub-lethal but stressful conditions that occur at the edges of a fixed temperature range in a fluctuating environment. By proactively adjusting temperature and dissolved oxygen in response to early stress behaviors, the system maintained the fish within a ‘preferred zone’ where energetic costs for thermal acclimation and stress response were minimized [44]. Changes in SOD and CAT activities, as well as MDA content, collectively reflect the degree of oxidative stress [45,46].

In the control group, the water temperature was set at 20 °C and dissolved oxygen at 5 mg/L—the latter being the critical level for maintaining normal life activities in fish. Dissolved oxygen fluctuates in actual aquaculture, easily inducing stress. The experimental group, through stress behavior feedback regulation, effectively reduced stress impact; whereas the control group, lacking real-time regulation capability, experienced greater stress. Persistent or frequent oxidative stress initially leads to an increase in the activity of related enzymes to counteract free radical damage. Over time, SOD and CAT activities gradually decline [47,48], while MDA content rises, indicating aggravated oxidative damage. Research has shown that appropriately increasing dissolved oxygen can alleviate the stress effects of adverse factors such as temperature [35,49]. By applying the multi-objective regulation strategy, the experimental group flexibly adjusted dissolved oxygen above 5 mg/L, optimizing the environment based on fish behavior feedback and growth performance, thereby effectively mitigating stress. This resulted in cortisol levels being maintained at a lower level, minimizing the overall stress impact. Although no significant differences were observed in final body weight, weight gain rate, or specific growth rate between the experimental and control groups, the control group lacked the capacity to respond to sudden stress events. In contrast, the experimental group—supported by the multi-objective regulation system—was able to promptly adapt to external changes and mitigate adverse environmental effects [50], thereby providing stable conditions conducive to fish growth. Furthermore, by consistently implementing the economically optimal regulation strategy, the experimental group achieved significantly lower energy consumption costs than the control group, demonstrating a more favorable economic profile. Note that the system validation experiment in this study lasted for 25 days, a timeframe sufficient to effectively evaluate the system’s core performance in terms of stress regulation response speed, short-term physiological adaptation, and cyclical economic benefits. However, it must be acknowledged that a 25-day period may not fully capture the growth performance and long-term physiological stability of aquaculture across its entire lifecycle. Although no significant differences in final weight and specific growth rate were observed between the experimental and control groups, a longer breeding cycle might amplify growth disparities caused by sustained low-intensity stress. Furthermore, the system’s long-term reliability requires further validation through extended experiments spanning the entire breeding season.

Although the system developed in this study has shown significant potential in optimizing economic benefits and fish welfare, there are still several limitations. Firstly, the system is currently based on a behavioral model and growth parameters of a single species (crucian carp) for regulation. The scalability and universality of its strategy on aquaculture species with different physiological characteristics or behavioral patterns (such as benthic fish or crustaceans) need to be verified. Secondly, the high-definition cameras, laser emitters, and precise temperature control equipment that the system relies on may result in high initial hardware costs, which may pose a certain economic threshold for small-scale farmers. In addition, system performance may be sensitive to breeding density, and under high-density conditions, fish occlusion may affect the accuracy of behavior recognition, thereby interfering with the precision of feedback control. In view of the above limitations, the future research work will focus on the following aspects, specifically, collecting the stress behavior data of various economic fishes, building a migratory and adaptive learning multi species behavior recognition model, exploring the deployment scheme of lightweight models based on low resolution cameras or edge computing equipment, to reduce the system hardware cost, introducing breeding density, individual size, etc. as dynamic variables into the multi-objective optimization function, developing more robust adaptive control strategies, and ensuring the stability and effectiveness of the system in complex production environments.

As indicated by the composite scores in Table 8, the initial group exhibited the least stress-induced damage, followed by the experimental group, while the control group showed the highest level of damage. By analyzing inter-variable correlations, this PCA model enabled a reliable and comprehensive assessment of stress-induced damage in crucian carp under aquaculture conditions, providing a valuable tool for health management in container-based fish farming systems.

## 5. Conclusions

This study has successfully developed and validated a multi-objective aquatic environmental regulation system based on stress behavior feedback. The system integrated the YOLOv8s-FasterNet behavior recognition model, specific growth rate model, and energy consumption cost model, with the objective of maximizing the “output-input ratio” to achieve a collaborative optimization of economic and welfare aspects in the breeding environment. During a 25-day experiment, the system achieved a cycle profit ranging from 1.03 to 8.93 times that of traditional threshold control methods, while ensuring no significant difference in growth compared to the control group. PCA confirmed that it significantly reduced stress-induced damage in crucian carp. The core value of this research lies in providing a scalable and precise intelligent decision-making framework for aquaculture. Although crucian carp was used as the model species in this study, the system’s technology is equally applicable to the farming of other fish species, such as tilapia and trout, which have different environmental requirements and economic values.

## Figures and Tables

**Table 1:** YOLOv8s-FasterNet network model performance indicators.

Behavior	Performance Metrics			
	mAP (%)	P (%)	R (%)	F1 (%)
Floating head behavior	98.40	98.60	95.20	96.87
Floating behavior	98.40	98.90	96.00	97.43
Rollover behavior	97.90	97.10	94.70	95.88

**Table 2:** Effect of multi-objective regulation strategy on growth parameters of crucian carp.

Groups	IF (g)	FW (g)	WGR (%)	SGR (%)	SR (%)
Control group	51.6 ± 1.3 ^a^	67.9 ± 1.2 ^a^	30.6 ± 2.8 ^a^	1.07 ± 0.09 ^a^	98.00
Experimental group	52.0 ± 0.8 ^a^	66.1 ± 2.0 ^a^	28.1 ± 3.1 ^a^	0.99 ± 0.10 ^a^	98.30

Different lowercase letters indicated significant differences among treatments (*p* < 0.05), n = 9.

**Table 3:** The energy consumption and economic benefits of the experimental group and the control group under the external condition of 2 °C.

Plan	KTemp	DDO	MTemp	MDO	Scost	Dcost	Sprofit	Dprofit
1	2	5	10	5	48.76	177.63	1.92	5.35
2	2	5	11	5	57.03	177.63	17.28	5.35
3	2	5	12	5	65.99	177.63	29.65	5.35
4	2	5	13	5	75.73	177.63	38.93	5.35
5	2	5	14	5	86.34	177.63	45.01	5.35
6	2	5	15	5	97.96	177.63	47.78	5.35
7	2	5	16	5	110.73	177.63	47.09	5.35
8	2	5	17	5	124.84	177.63	42.74	5.35
9	2	5	18	5	140.50	177.63	34.53	5.35
10	2	5	19	5	157.98	177.63	22.18	5.35
11	2	5	20	5	177.63	177.63	5.35	5.35
12	2	5	21	5	199.88	177.63	−16.39	5.35
13	2	5	22	5	225.27	177.63	−43.58	5.35
14	2	5	23	5	254.52	177.63	−76.95	5.35
15	2	5	24	5	288.59	177.63	−117.45	5.35
16	2	5	25	5	328.78	177.63	−166.38	5.35
17	2	5	26	5	376.88	177.63	−225.54	5.35
18	2	5	27	5	435.49	177.63	−297.52	5.35
19	2	5	28	5	508.50	177.63	−386.21	5.35
20	2	5	29	5	601.93	177.63	−497.64	5.35
21	2	5	30	5	725.76	177.63	−641.77	5.35

KTemp: current temperature; DDO: current dissolved oxygen; MTemp: target temperature; MDO: target dissolved oxygen; Scost: energy consumption cost of the experimental group; Dcost: energy consumption cost of the control group; Sprofit: profit of the experimental group; Dprofit: profit of the control group.

**Table 4:** The energy consumption and economic benefits of the experimental group and the control group under the external condition of 8 °C.

Plan	KTemp	DDO	MTemp	MDO	Scost	Dcost	Sprofit	Dprofit
1	8	5	10	5	9.91	86.34	40.76	96.64
2	8	5	11	5	15.35	86.34	58.96	96.64
3	8	5	12	5	21.14	86.34	74.50	96.64
4	8	5	13	5	27.34	86.34	87.32	96.64
5	8	5	14	5	33.97	86.34	97.39	96.64
6	8	5	15	5	41.09	86.34	104.65	96.64
7	8	5	16	5	48.76	86.34	109.06	96.64
8	8	5	17	5	57.03	86.34	110.55	96.64
9	8	5	18	5	65.99	86.34	109.04	96.64
10	8	5	19	5	75.73	86.34	104.44	96.64
11	8	5	20	5	86.34	86.34	96.64	96.64
12	8	5	21	5	97.96	86.34	85.53	96.64
13	8	5	22	5	110.73	86.34	70.96	96.64
14	8	5	23	5	124.84	86.34	52.74	96.64
15	8	5	24	5	140.50	86.34	30.65	96.64
16	8	5	25	5	157.98	86.34	4.42	96.64
17	8	5	26	5	177.63	86.34	−26.29	96.64
18	8	5	27	5	199.88	86.34	−61.91	96.64
19	8	5	28	5	225.27	86.34	−102.98	96.64
20	8	5	29	5	254.52	86.34	−150.23	96.64
21	8	5	30	5	288.59	86.34	−204.61	96.64

**Table 5:** The energy consumption and economic benefits of the experimental group and the control group under the external condition of 14 °C.

Plan	KTemp	DDO	MTemp	MDO	Scost	Dcost	Sprofit	Dprofit
1	14	5	14	5	0.00	33.97	131.36	149.02
2	14	5	15	5	4.81	33.97	140.94	149.02
3	14	5	16	5	9.91	33.97	147.90	149.02
4	14	5	17	5	15.35	33.97	152.23	149.02
5	14	5	18	5	21.14	33.97	153.88	149.02
6	14	5	19	5	27.34	33.97	152.83	149.02
7	14	5	20	5	33.97	33.97	149.02	149.02
8	14	5	21	5	41.09	33.97	142.40	149.02
9	14	5	22	5	48.76	33.97	132.93	149.02
10	14	5	23	5	57.03	33.97	120.54	149.02
11	14	5	24	5	65.99	33.97	105.15	149.02
12	14	5	25	5	75.73	33.97	86.67	149.02
13	14	5	26	5	86.34	33.97	65.00	149.02
14	14	5	27	5	97.96	33.97	40.01	149.02
15	14	5	28	5	110.73	33.97	11.56	149.02
16	14	5	29	5	124.84	33.97	−20.54	149.02
17	14	5	30	5	140.50	33.97	−56.51	149.02

**Table 6:** Total variance solved.

Ingredient	Initial Eigenvalue			Extract the Sum of Squares and Load It		
	Total	Variance/%	Cumulation/%	Total	Variance/%	Cumulation/%
$F_{1}$	2.452	49.045	49.045	2.452	49.045	49.045
$F_{2}$	1.425	28.495	77.540	1.425	28.495	77.540
$F_{3}$	0.497	9.930	87.470			
$F_{4}$	0.333	6.669	94.140			
$F_{5}$	0.293	5.860	100.000			

**Table 7:** Principal Component Load Matrix.

Correlation Coefficient	Ingredient	
	$F_{1}$	$F_{2}$
$\mathrm{LZM}\;\mathrm{activity}\;(X_{1}$)	−0.089	−0.513
$\mathrm{SOD}\;\mathrm{activity}\;(X_{2}$)	0.480	0.285
$\mathrm{CAT}\;\mathrm{activity}\;(X_{3}$)	0.378	−0.060
$\mathrm{Cortisol}\;\mathrm{level}\;(X_{4}$)	0.050	0.488
$\mathrm{MDA}\;\mathrm{content}\;(X_{5}$)	−0.351	0.128

**Table 8:** Comprehensive Score and Sorting.

Groups	Overall Score	Ranking
Initial group (A)	0.223	1
Experimental group (B)	−0.036	2
Control group (C)	−0.348	3

## Data Availability

The original contributions presented in the study are included in the article, further inquiries can be directed to the corresponding author.
